# Supplementary material for: H3K4me1 Modification Functions in Caste Differentiation in Honey Bees
Source: Int J Mol Sci. 2023 Mar 25;24(7):6217. doi: 10.3390/ijms24076217 (PMC10094490; doi:10.3390/ijms24076217)
Supplement: Supplementary file 1 [file ijms-24-06217-s001.zip › Figure S1-S6; Table S1.pdf]

Supplemental Materials

H3K4me1 Modification Functions in Caste Differentiation in Honey Bees

Yong Zhang [1,2,†], Zhen Li [1,2,†], Xujiang He [1,2], Zilong Wang [1,2] and Zhijiang Zeng [1,2,*]

[1] Honeybee Research Institute, Jiangxi Agricultural University, Nanchang 330045, China
[2] Jiangxi Province Key Laboratory of Honeybee Biology and Beekeeping, Nanchang 330045, China
* Correspondence: bees@jxau.edu.cn
† These authors contributed equally to this work.

Table of contents

Supplemental Figures S1 – S6

Supplemental Tables S1

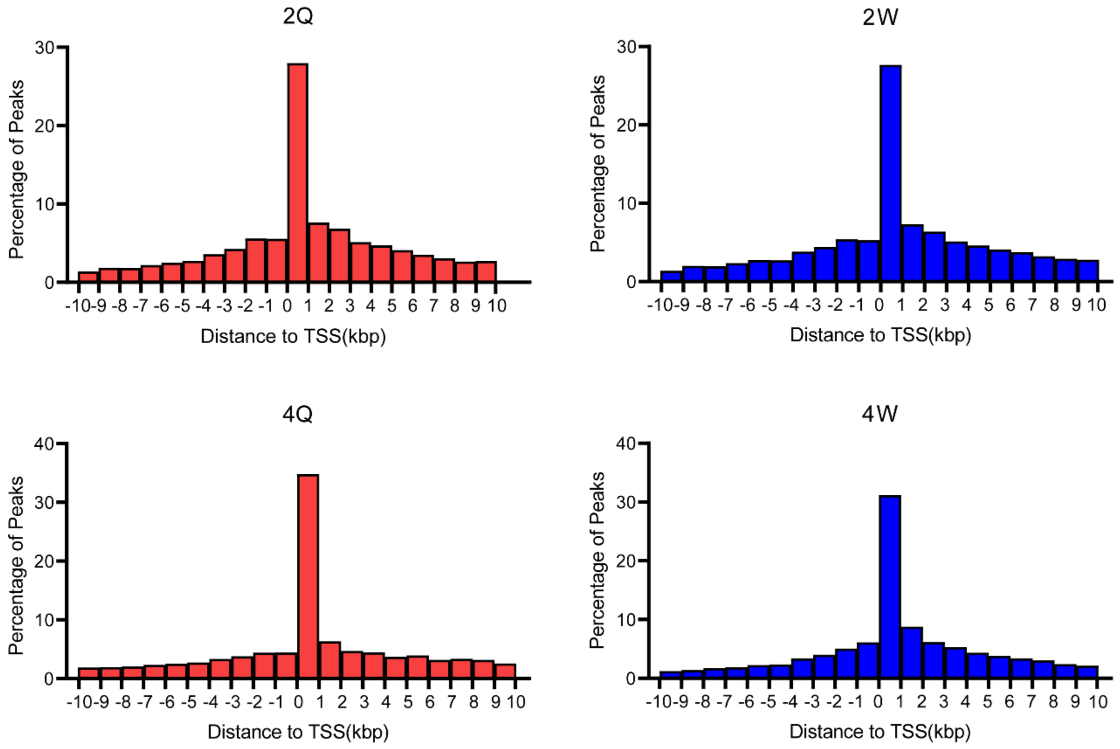

Figure S1. Bar plots showing the percentage of H3K4me3 peaks that the distance from the nearest TSS. This revealed that over 50% of all H3K4me1 peaks are located within +/- 2kbp of the TSS of a gene.

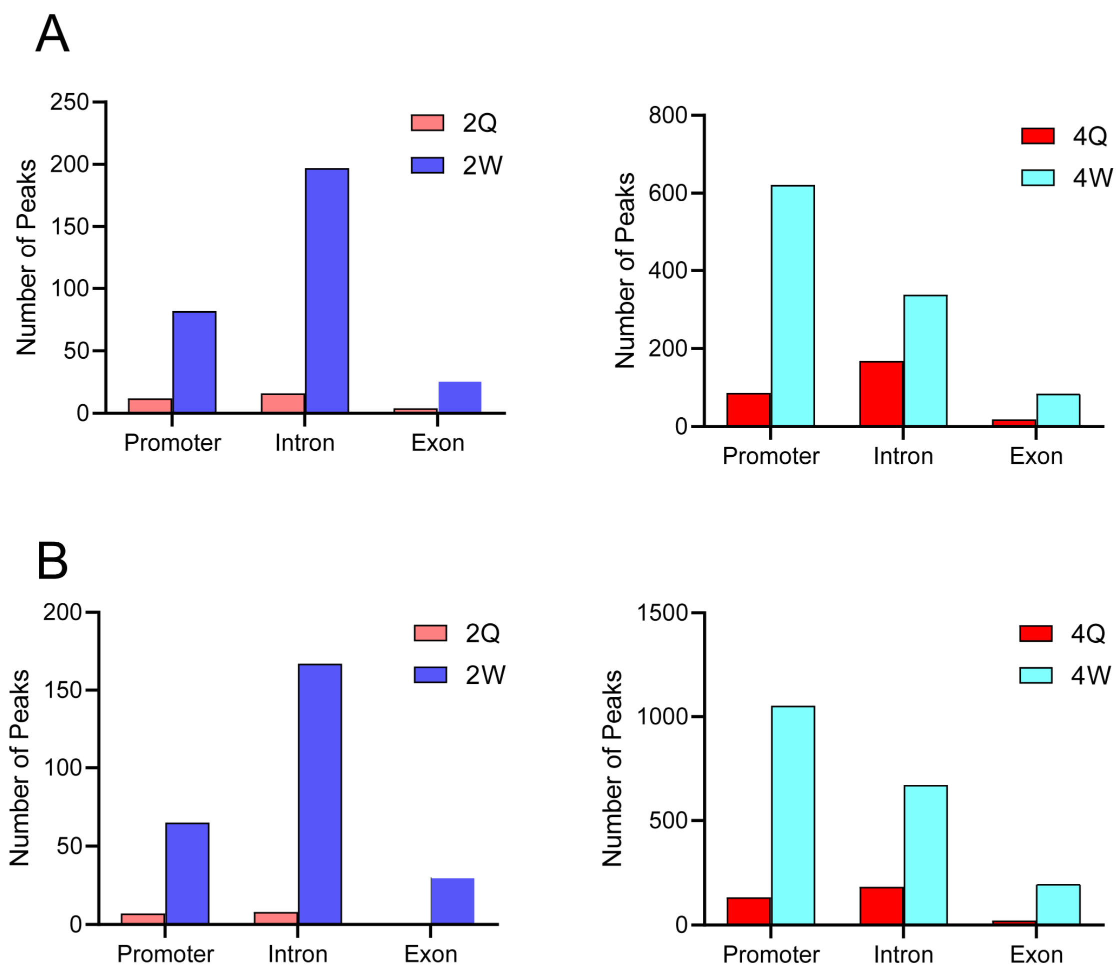

Figure S2. (**A**) A bar plot showing the number of unique H3K4me1 ChIP-seq regions within promoter, intron or exon in 2Q vs 2W and 4Q vs 4W. (**B**) A bar plot showing the number of differential H3K4me1 ChIP-seq regions within promoter, intron or exon in 2Q vs 2W and 4Q vs 4W.

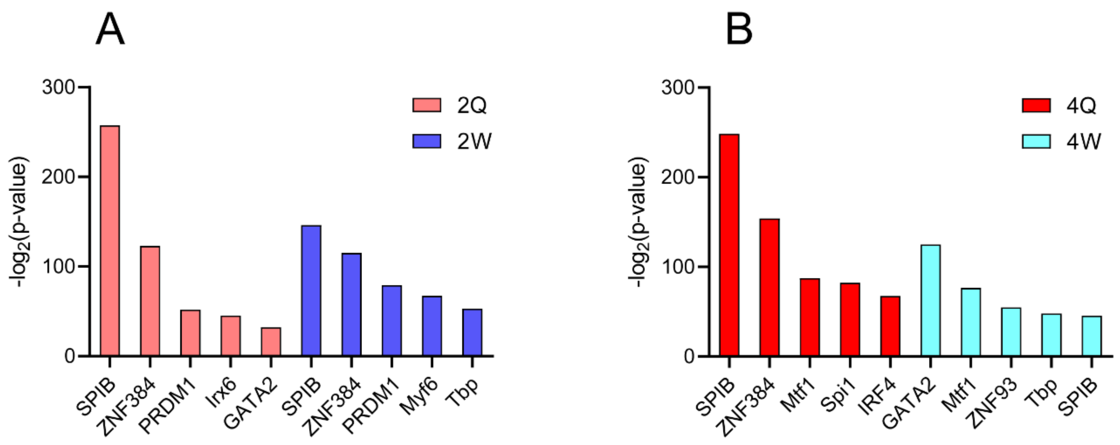

Figure S3. (**A**) A bar plot of the P-value for enrichment of transcription factor binding

motifs located within caste-specific promoter H3K4me1 regions in 2Q and 2W. (B) A bar plot of the P-value for enrichment of transcription factor binding motifs located within caste-specific promoter H3K4me1 regions in 4Q and 4W.

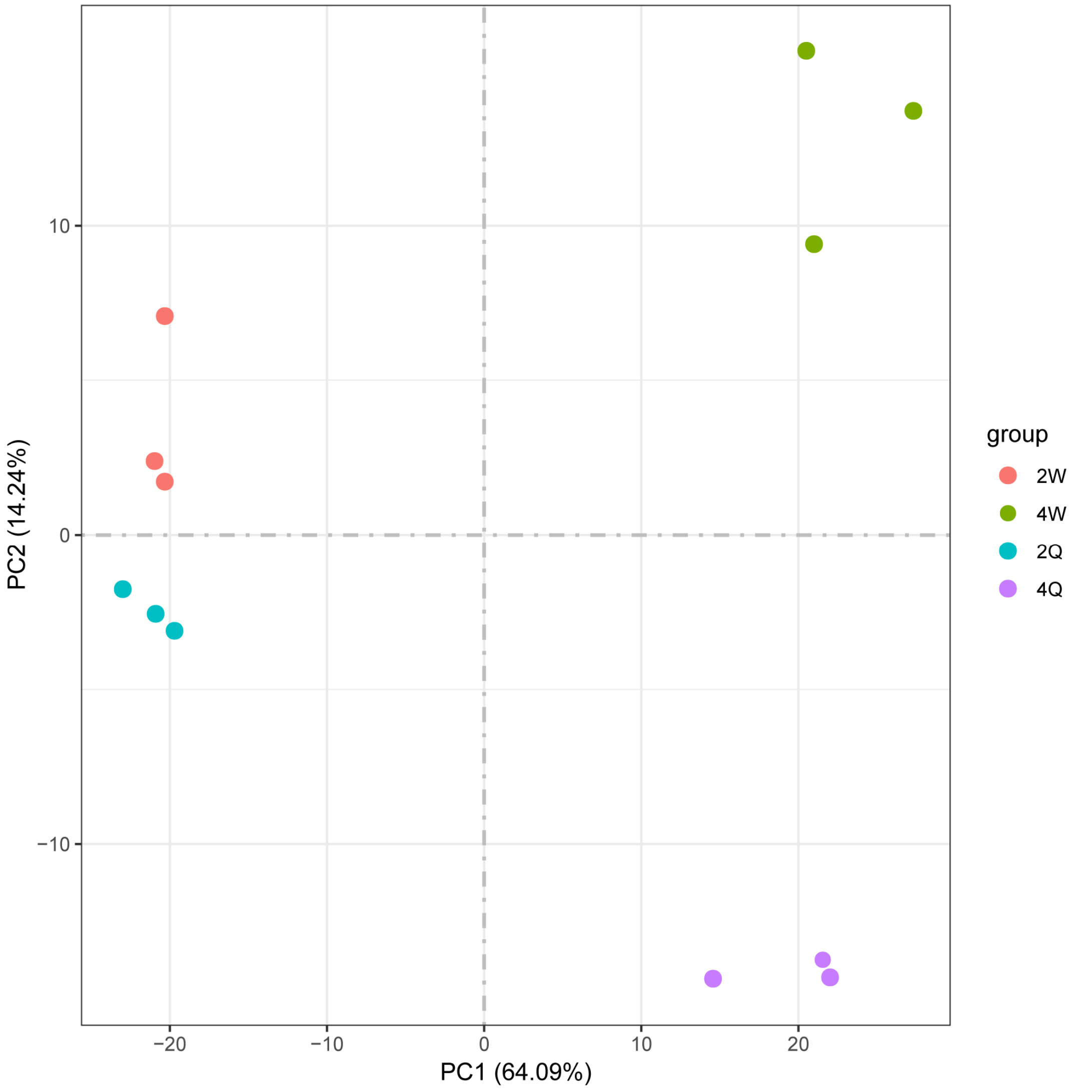

Figure S4. A multidimensional scaling plot of the gene expression values as measured by RNA-seq for 2Q, 2W, 4Q and 4W.

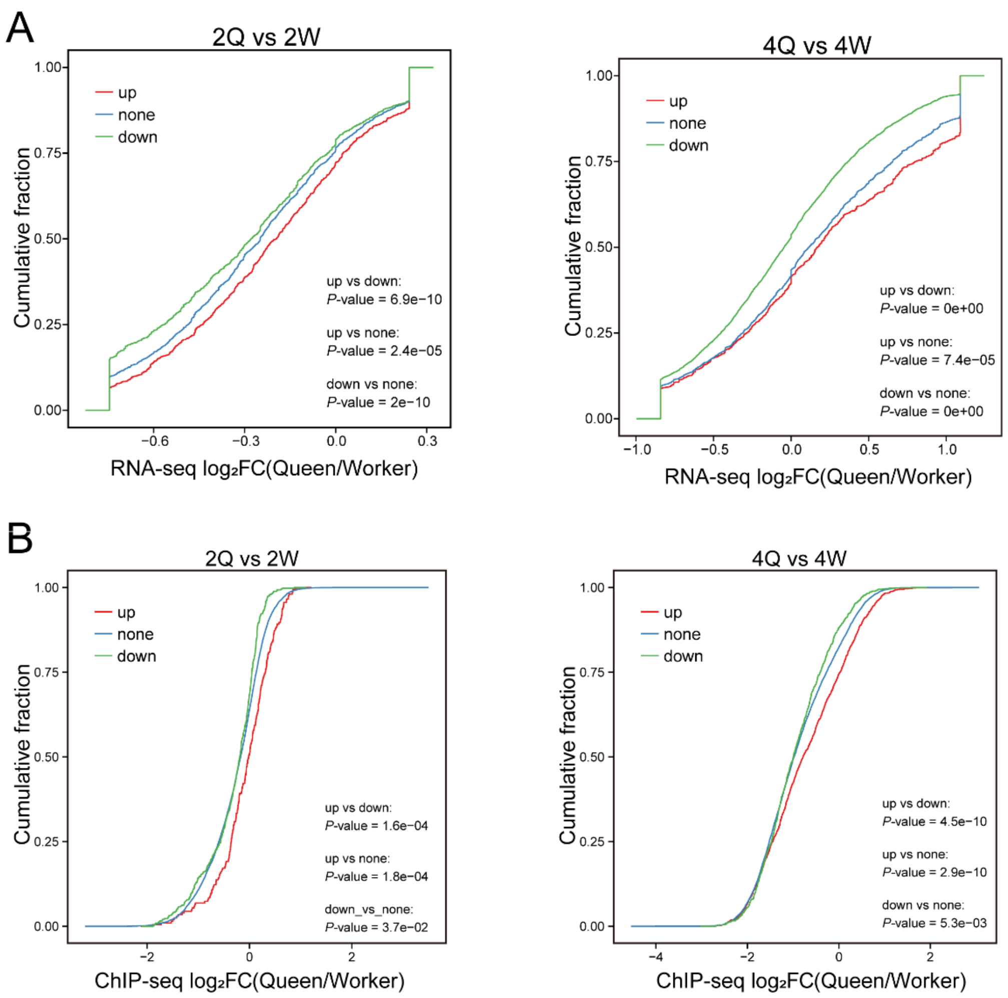

Figure S5. (**A**) For the differentially up-regulated, down-regulated and non-differentially expressed genes from RNA-seq data, the ChIP-seq RPM values of their promoter regions in the two groups of experiments were calculated, log2(fold change) was calculated, and the cumulative distribution map was used. Test by Kolmogorov-Smirnov test. (**B**) For the differentially up-regulated, down-regulated and non-differentially up-regulated peaks of each comparison combination, their corresponding genes were found, and the cumulative distribution map of the log2 (fold change) of RNA-seq FPKM of these genes was made. Test by Kolmogorov-Smirnov test.

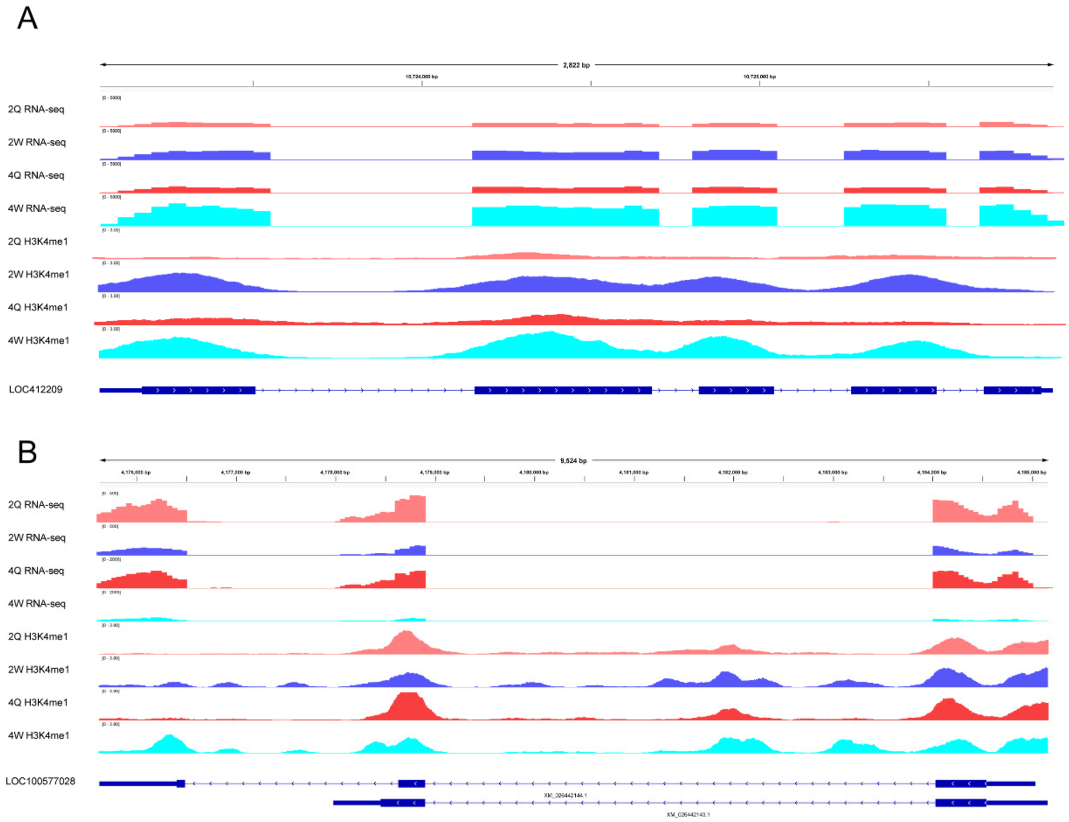

Figure S6. (**A**) A screenshot from the genome browser display showing the read alignments over probable cytochrome P450 6a17 (*P450-6a17*; LOC412209). (**B**) A screenshot from the genome browser display showing the read alignments over insulin-like growth factor I (*IGF*; LOC100577028).

Table S1. Detailed mapping statistics for ChIP-seq.

Sample	Histone	Replicate	Sequenced Reads	Mapped Reads	Percentage	Q30
2Q	H3K4me1	R1	14631419	13262767	90.65%	91.12%
		R2	10583044	9567337	90.40%	90.90%
		R3	10059597	9165651	91.11%	92.11%
2W	H3K4me1	R1	10144828	9358660	92.25%	90.41%
		R2	12149625	10984726	90.41%	91.56%
		R3	14487201	13255502	91.50%	92.02%
4Q	H3K4me1	R1	8215040	8138640	99.07%	91.06%

		R2	10302880	9680390	93.96%	92.05%
		R3	12536409	11765869	93.85%	91.72%
4W	H3K4me1	R1	10332269	10188373	98.61%	91.83%
		R2	9930141	9761188	98.30%	92.72%
		R3	9937744	9798071	98.59%	91.92%
2Q	Input	R1	11355197	11192155	98.56%	91.95%
		R2	11488832	11340855	98.71%	91.12%
		R3	12138169	11722977	96.58%	90.90%
2W	Input	R1	11761430	11578360	98.44%	92.67%
		R2	11625109	11379636	97.89%	90.41%
		R3	15888358	15595169	98.15%	91.56%
4Q	Input	R1	11800077	11325868	95.98%	92.35%
		R2	10938533	10786943	98.61%	91.06%
		R3	11651392	11497130	98.68%	92.05%
4W	Input	R1	14019768	13709187	97.78%	92.22%
		R2	12512339	12166176	97.23%	91.83%
		R3	11241926	10978466	97.66%	92.72%
